# Pectin methylesterases contribute the pathogenic differences between races 1 and 4 of *Fusarium oxysporum* f. sp. *cubense*

**DOI:** 10.1038/s41598-017-13625-4

**Published:** 2017-10-13

**Authors:** Huiyun Fan, Honghong Dong, Chunxiang Xu, Jing Liu, Bei Hu, Jingwen Ye, Guiwan Mai, Huaping Li

**Affiliations:** 1 State Key Laboratory of Conservation and Utilization of Subtropical Agro-bioresources, Guangdong Province Key Laboratory of Microbial Signals and Disease Control, College of Agriculture, South China Agricultural University, Guangzhou, 510642 China; 2 College of Horticulture, South China Agricultural University, Guangzhou, 510642 China

## Abstract

Plant cell walls, which are mainly composed of pectin, play important roles in plant defence responses to pathogens. Pectin is synthesised in a highly esterified form and then de-esterified by pectin methylesterases (PMEs). Because of this, PMEs are directly involved in plant defence. However, the molecular mechanisms of their interactions with pectins remain unclear. In this study, we compared the expression level and enzyme activities of PMEs in a banana Cavendish cultivar (*Musa* AAA ‘Brazilian’) inoculated with *Fusarium oxysporum* f. sp. *cubense* pathogenic races 1 (*Foc*1) and 4 (*Foc*4). We further examined the spatial distribution of PMEs and five individual homogalacturonans (HGs) with different degree of pectin methylesterification (DM). Results suggested that the banana roots infected with *Foc*1 showed lower PME activity than those infected with *Foc*4, which was consisted with observed higher level of pectin DM. The level of HGs crosslinked with Ca^2+^ was significantly higher in roots infected with *Foc*1 compared with those infected with *Foc*4. Therefore, banana exhibited significantly different responses to *Foc*1 and *Foc*4 infection, and these results suggest differences in PME activities, DM of pectin and Ca^2+^-bridged HG production. These differences could have resulted in observed differences in virulence between *Foc*1 and *Foc*4.

## Introduction

*Fusarium oxysporum* f. sp. *cubense* (*Foc*), the causal agent of banana *Fusarium* wilt, can cause severe losses in yield and quality. The pathogen penetrates banana roots, colonises and blocks vascular tissues, causes a reddish-brown discolouration of the rhizome and pseudostem, and eventually leads to leaf collapse and plant death^1^. Susceptible banana plants can only be grown in pathogen-free soil because *Foc* can survive in the soil for decades^2^. According to the ability to cause disease to certain cultivars in the field, pathogenic variability within *Foc* causes its subdivision into races. Three races (1, 2 and 4) of the pathogen affect banana. *Foc* race 1 (*Foc*1) attacks dessert bananas, such as ‘Gros Michel’ (*Musa* spp., AAA-group), and caused the 20th century epidemic resulting in the inability to grow that cultivar for the mass market. *Foc* race 2 (*Foc*2) affects ‘Bluggoe’ (*Musa* spp., ABB-group) and its closely related cultivars. *Foc* race 4 (*Foc*4) causes disease to most commercially grown ‘Cavendish’ banana cultivars (*Musa* spp., AAA-group), as well as the hosts of *Foc*1 and *Foc*2.

Cell walls serve as external barriers to prevent pathogens from infecting or spreading within a plant. Homogalacturonans (HGs) are the main constituents of cell wall pectin, and therefore play a crucial role in the first defence against pathogen attack^3^. Necrotrophic fungi usually degrade pectin to achieve a successful infection^4^. *F*. *oxysporum*, a hemibiotrophic and/or necrotrophic pathogen, releases a series of cell wall-degrading enzymes (CWDEs) that digest host plant cell walls^3,5^. The pectin methylesterases (PMEs), one of the CWDEs, remove methyl esters from pectin and improve the cell wall accessibility to other CWDEs^6,7^.

A series of strategies, such as hydroxylation of plant cell wall components and production of inhibitor proteins, is used by plants against pathogenic CWDEs to prevent degradation of pectin^8,9^. Plants finely tune pectin methylesterification and regulate PME activities during normal growth and development. In plant–pathogen interactions, the activities of PMEs^10^, level of PMEs inhibitor (PMEI)^9^, degree of methylesterfication (DM) of HGs^11^ and molecular properties of HGs^8^ are associated with plant resistance to pathogens. The extensive degradation of HGs by pathogens causes the production of oligogalacturonides (OGs) that act as elicitors of defence responses in the host^3,12^ including changes in the expression of defence-associated genes^12^. However, during the interaction between plant and pathogen, the specific roles of PMEs and molecular mechanisms of PMEs action on pectin remain unclear, and conflicting results also were occasionally reported about the level of PME expression^5,13^, and changes in cell wall composition^14,15^ that occurs when different plants species are infected by different pathogens.

During the interaction between banana and *Foc*, both of *Foc*1 and *Foc*4 can infect ‘Brazilian’ banana, but only *Foc*4 can cause ‘Brazilian’ banana disease^16^. To date, most of the research work about *Foc* has focused on molecular detection^17,18^, pathogenesis-related genes^19^, the infection process of *Foc*4^20^, resistance-related genes of host^21^, and disease control^22,23^. There was a report that the spatial distributions of PMEs and different pectin DM affect susceptibility of different banana cultivars to *Foc*4^24^. Nevertheless, no study addressed the differences in pathogenicity between *Foc*1 and *Foc*4 using cell histological observations. In addition, whether and how PMEs affect differences in observed virulence between pathogen races remains unclear.

In the present study, we mainly focused on examining whether there are differences in the enzyme activities of PMEs and DMs of HGs in ‘Brazilian’ banana challenged with *Foc*1 and *Foc*4. Furthermore, whether or not PMEs affect the pathogenicity of *Foc*1 and *Foc*4 on ‘Brazilian’ banana also was addressed. To this end, the enzyme activities and expression of *PME*1 of *Musa accuminata* (*MaPME1*) gene were compared in ‘Brazilian’ banana challenged with *Foc*1 and *Foc*4. Likewise, observations of spatial distributions of PMEs and pectin with different DMs were made, and the relationship with of these obseversations with observed pathogenicity of *Foc*1 and *Foc*4 was determined by measuring the abundance of *Foc* in plants inoculated by *Foc*, as well as the expression level of six defence genes in roots treated with OGs, *Foc*1 and *Foc*4. Based on our results, we could make conclusions whether or not plant PME activities, methyl-esterified levels of pectin, relatively DM of pectin, and production of HG-bridged by Ca^2+^ could impact pathogenicity of different *Foc* races on ‘Brazilian’ banana.

## Results

### Differences in the activities of PMEs and the DMs of pectin

**Figure 1:**
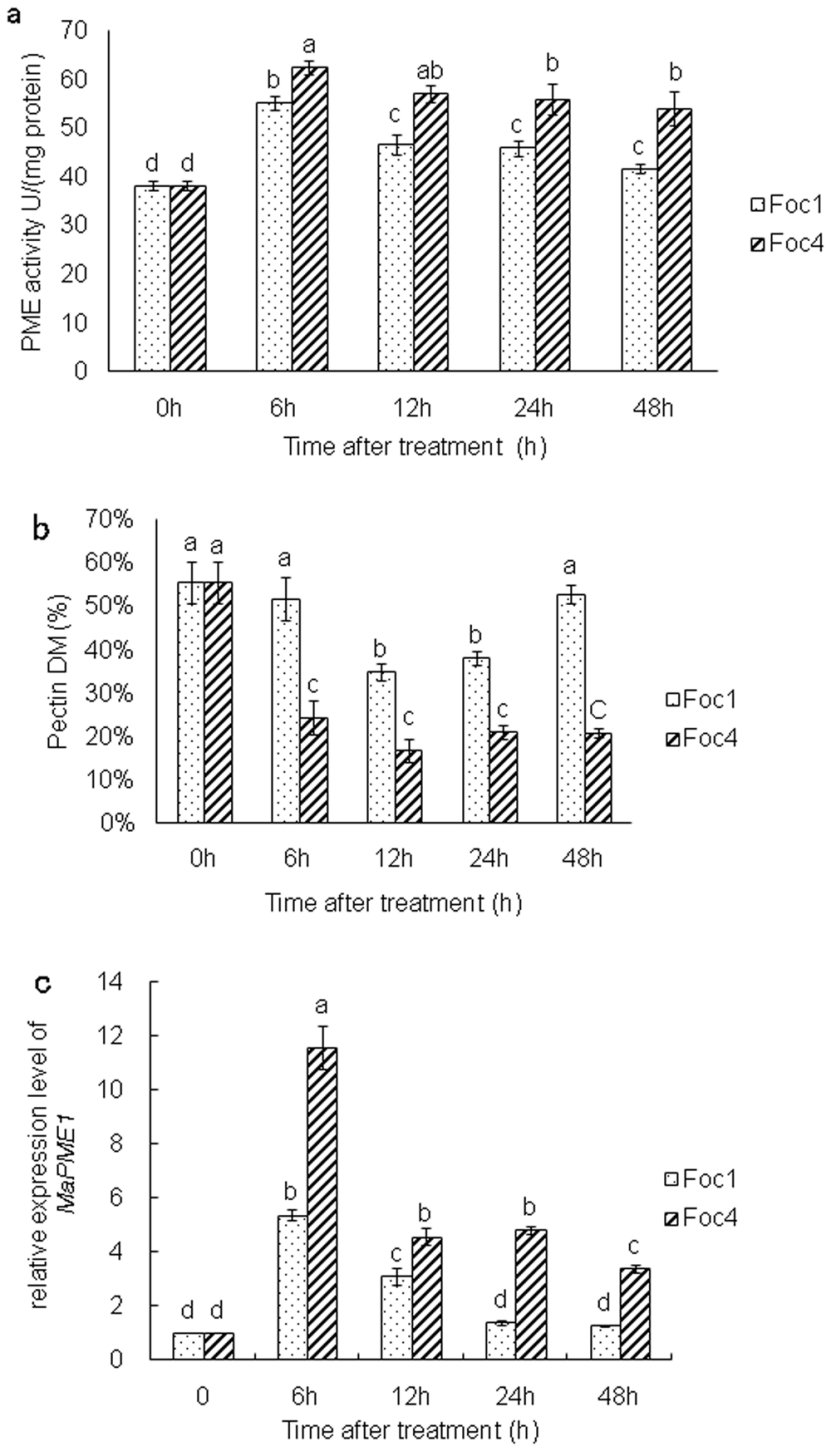
The changes in PME activities (**a**), pectin DMs (**b**) and in transcript levels of *MaPME1* (**c**)in banana (*Musa* spp. AAA) roots different hours after infection with *Foc*1 and *Foc*4 of *Fusarium oxysporum* f. sp. *cubense*, respectively. Data represent an average of three replicates ± SD. Values followed by the same letter are not significantly different using Duncan’s multiple range test at P < 0.05 after angular transformation of the data. Data of the DMs were expressed as the ratio of mean methanol content (in moles; n = 3) to 1 mol GalA (n = 3).

The PME activity of the roots infected with *Foc*1 was remarkably lower than that of the roots inoculated with *Foc*4 (Fig. 1a). However, the general change of PME activities in roots inoculated respectively with both *Foc*1 and *Foc*4 was similar. In brief, the PME activities increased from 0 to 6 h, and kept in a lower and stable state from 12 h to 48 h. However, the PME activities were still higher at 48 h after infection than those of controls.

The DM of pectin in the roots inoculated with *Foc*1 was significantly higher compared with those of roots inoculated with *Foc*4. The DM of the roots inoculated with *Foc*1 showed no considerable change at 6 h, decreased markedly from 12 h to 24 h after infection and finally almost restored the level of the controls. By contrast, the DM of the roots inoculated with *Foc*4 considerably decreased at 6 h, and maintained a lower level in any other time point (Fig. 1b). The higher DM in the roots infected with *Foc*1 coincided with low PME activity. In addition, the DM of the roots infected with *Foc*1 represented an almost 3-fold difference in the DM of roots inoculated with *Foc*4.

### Changes in relative expression levels of *MaPME1*

We investigated the transcript levels of *MaPME1* to confirm the results of PME activity measurements by qRT-PCR. The amplification of the standard dilution series yielded linear and reliable results (*MaPME1*: R^2^ = 0.993; *RPS2*: R^2^ = 0.979), as well as the high efficiency of the amplification (*MaPME1*: Eff = 100.2%; *RPS2*: Eff = 100.1%). The *MaPME1* showed significantly different expression at any time point in the roots infected with *Foc*1 compared with the roots inoculated with *Foc*4 (Fig. 1c). The qRT-PCR validation of this gene was in agreement with the above study. The transcript levels of *MaPME1* were highest at 6 h in the roots infected with the two pathogenic races, but the transcript level of *MaPME1* in roots inoculated with *Foc*4 was twice as high as that in roots infected with *Foc*1. The transcript level of *MaPME1* in the roots infected with *Foc*1 decreased from 12 h to 24 h and then returned to level similar to controls. Nevertheless, the transcript level of *MaPME1* in the roots inoculated with *Foc*4 showed a decrease after 12 h, no changes from 12 h to 24 h, and decreased expression after 48 h. In general, the transcript level of *MaPME1* in the roots inoculated with *Foc*4 was greater than that in the roots infected with *Foc*1 at all time-points.

### Spatial and temporal distribution of PMEs

**Figure 2:**
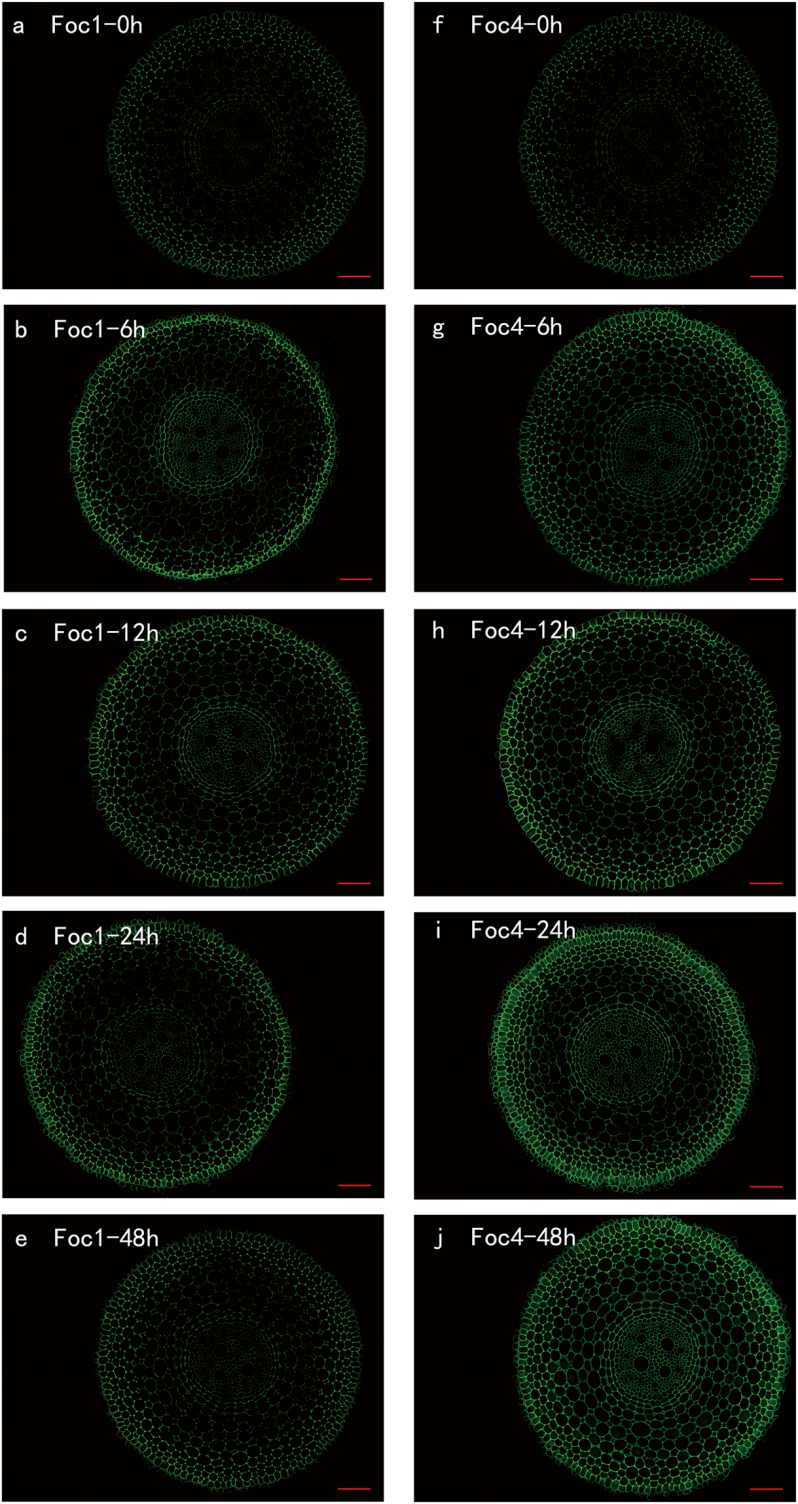
Immunolocalization by a pan-specific PME antibody recognizing more PMEs isoforms (**a**–**j**) in banana (*Musa* spp. AAA) roots at different hours (0, 6, 12, 24, 48 h) after infection with *Foc*1 and *Foc*4. The banana roots infected by *Foc*1: (**a**–**e**); the banana roots infected by *Foc*4: (**f**–**j**) The immunolabelling observed before the treatment (**a**,**f**), 6 hours after infection (**b**,**g**), 12 hours after infection (**c**,**h**), 24 hours after infection (**d**,**i**) and 48 hours after infection (**e**,**j**) are presented. Bars represent 100 µm.

Before the treatment with pathogens, strong signals were observed in the epidermis, endodermis and cortical cells close to the epidermis; by contrast, weak signals emerged in the most cortical cells and vascular cylinder (Fig. 2a,f). PME immunolabelling of roots infected with *Foc*1 was lower than that of roots infected with *Foc*4 during the entire experiment. First, the labelling in the roots inoculated with *Foc*1 and *Foc*4 increased mainly in the epidermis, endodermis and steles at 6 h after infection (Fig. 2b, g). Afterwards, the level of epitope decreased slowly from 12 h to 48 h in roots infected with *Foc*1, particularly in epidermis and stele (Fig. 2c–e), but that of roots inoculated with *Foc*4 showed sharp increase at all the times, thereby the strong signals observed in the whole root sections at 48 h (Fig. 2g–j). Moreover, the signals of roots inoculated with *Foc*4 were fourfold higher than those of roots infected with *Foc*1 (see Supplementary Fig. S1). The results indicated that the variation trends were similar to those in the above experiments. These results might mean that higher activities of PMEs resulted in the stronger pathogenicity of *Foc*4 compared with that of *Foc*1 in ‘Brazilian’ banana.

### Changes in subcellular distribution of HGs with different DMs during host–pathogen interactions

We used several antibodies, which bound different degrees of methyl-esterified HGs, to monitor the dynamic changes in the subcellular distribution of HGs with different DMs during host–pathogen interactions and subsequently understand the mechanism of how PMEs modify pectin and how the subcellular distribution of HGs with different DMs changes during host–pathogen interactions.

### LM20

**Figure 3:**
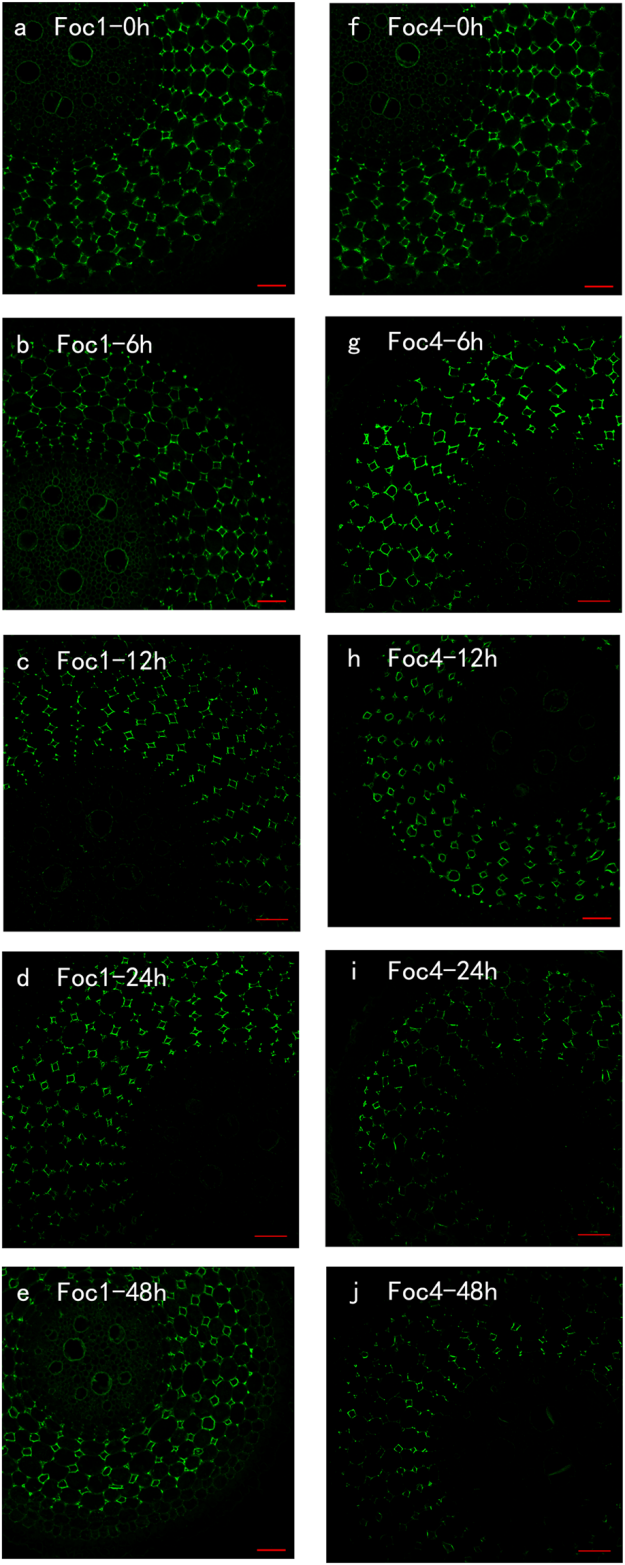
The changes in immunolocalization by LM20 antibody in banana (*Musa* spp. AAA) roots at different hours (0, 6, 12, 24, 48 h) after infection by *Fusarium oxysporum* f. sp. *cubense*. The banana roots infected by *Foc*1: (**a**–**e**); the banana roots infected by *Foc*4: (**f**–**j**). The immunolabelling observed before the treatment (**a**,**f**), 6 hours after infection (**b**,**g**), 12 hours after infection (**c**,**h**), 24 hours after infection (**d**,**i**) and 48 hours after infection (**e**,**j**) are presented. Bars represent 50 µm.

LM20 antibody recognises highly methyl-esterified HGs. After the roots were treated with *Foc*, the changes in labelling were mainly observed in the cortical cells and steles. The binding of LM20 was stronger in the roots infected with *Foc*1 than in the roots infected with *Foc*4 (Fig. 3a–j). These results were consistent with the results of PME activities. During the co-incubation of the roots with *Foc*1, the signal reduced in the steles and then in the cortical cells at 6 and 12 h after infection (Fig. 3a–c); starting from 24 h, the antigen levels showed an upward trend and almost recovered the level of the control. By contrast, the abundance of antigen decreased almost linearly in the roots inoculated with *Foc*4 (Fig. 3f–j). Additionally, the level of antigen in roots inoculated with *Foc*1 was fourfold of that in roots inoculated with *Foc*4 after 48 h (Fig. 3e,j; see Supplementary Fig. S2). These two treatments presented no substantial difference before 12 h, but a crosscurrent from 24 h to 48 h (Fig. 3d,e,i,j; see Supplementary Fig. S2).

### LM19

**Figure 4:**
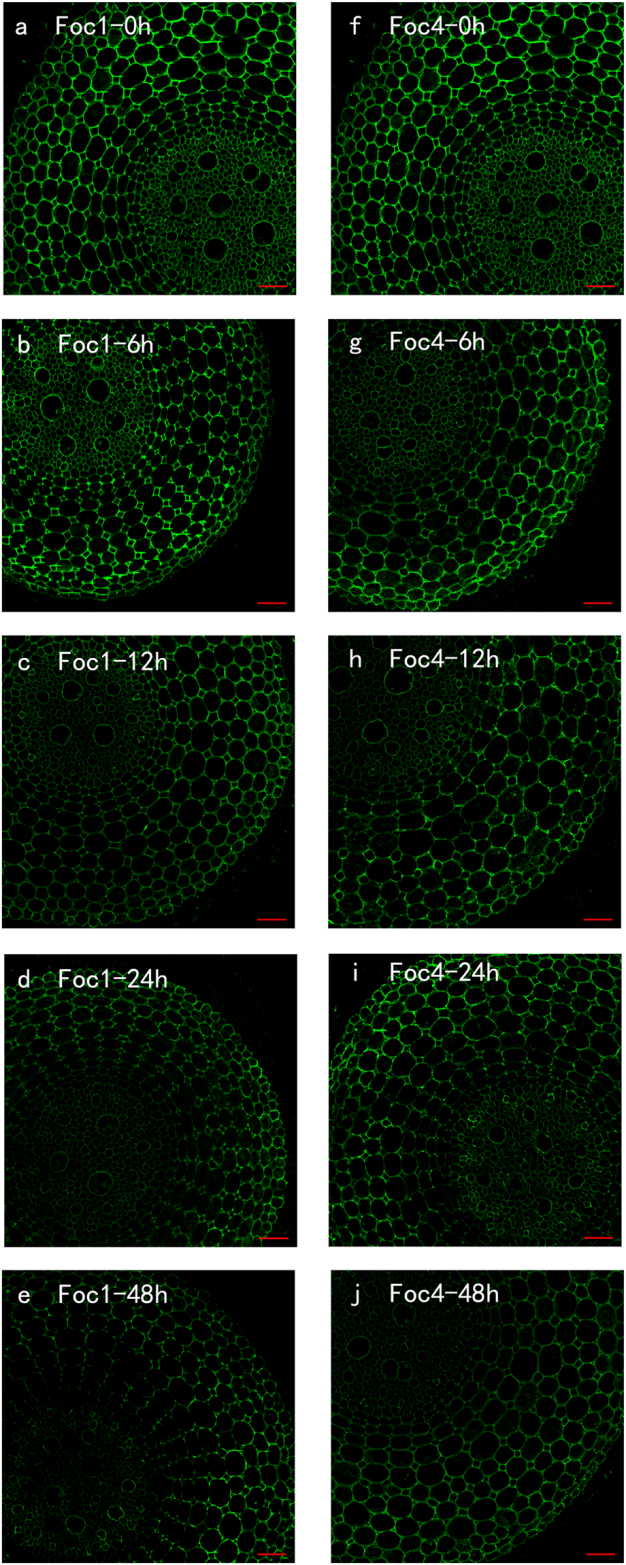
The changes in immunolocalization by LM19 antibody in banana (*Musa* spp. AAA) roots at different hours (0, 6, 12, 24, 48 h) infection by *Fusarium oxysporum* f. sp. *cubense*. The banana roots infected by *Foc*1: (**a**–**e**); the banana roots infected by *Foc*4: (**f**–**j**). The immunolabelling observed before the treatment (**a**,**f**), 6 hours after infection (**b**,**g**), 12 hours after infection (**c**,**h**), 24 hours after infection (**d**,**i**) and 48 hours after infection (**e**,**j**) are presented. Bars represent 50 µm.

LM19 antibody binds low methyl-esterified or demethyl-esterified HGs. LM19 immunolabelling was evenly distributed over the whole root section when the roots were non-treated with *Foc* (Fig. 4a,f). The labelling, in the roots after 6 h of infection with *Foc*, was less intense compared with the controls, and no considerable difference was observed (Fig. 4b,g). However, the corresponding antigen of roots inoculated with *Foc*4 was higher than that of roots infected with *Foc*1 from 12 h to 48 h (Fig. 4c–e and h–j). From the perspective of fluorescence signal intensity, the binding of LM19 in roots inoculated with *Foc*4 was twice less after 48 h than that of mock inoculation. Furthermore, the binding of LM19 in roots infected with *Foc*1 was fourfold lower than that of mock inoculation (see Supplementary Fig. S3).

### JIM7

**Figure 5:**
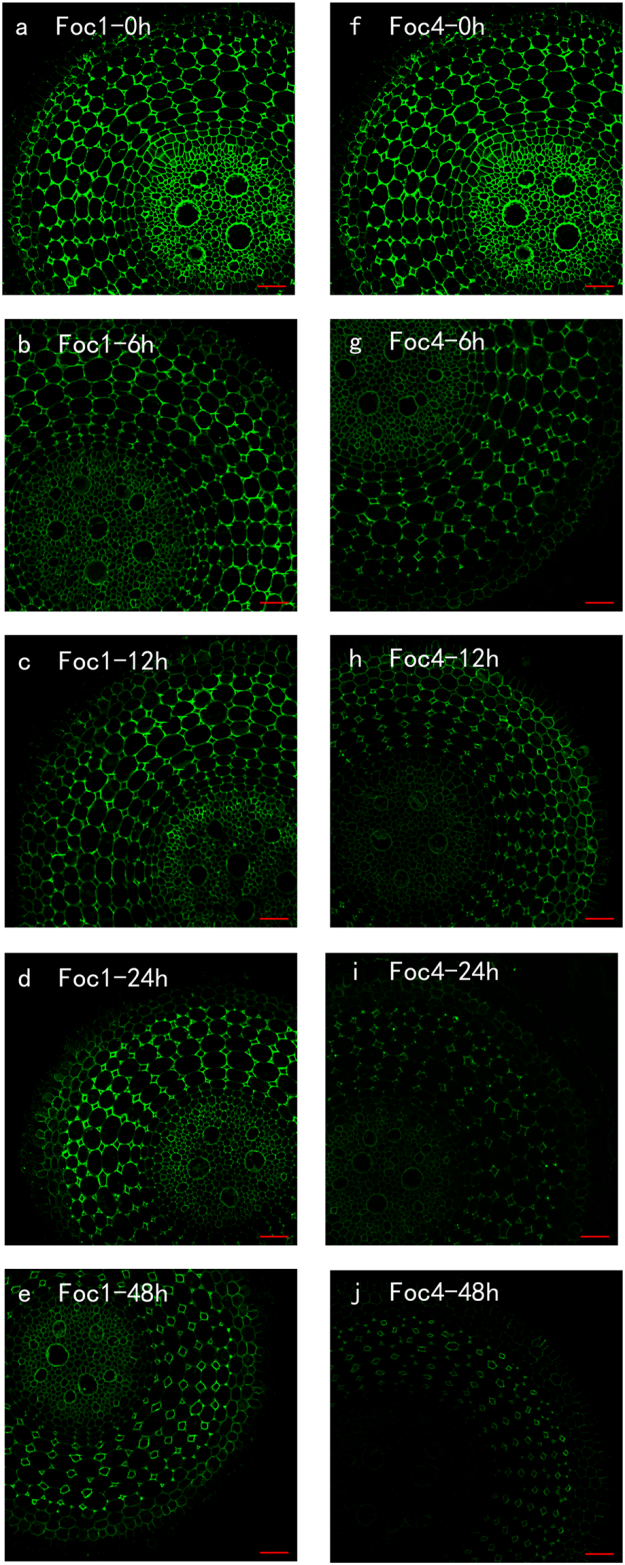
The changes in immunolocalization by JIM7 antibody in banana (*Musa* spp. AAA) roots at different hours (0, 6, 12, 24, 48 h) infection by *Fusarium oxysporum* f. sp. *cubense*. The banana roots infected by *Foc*1: (**a**–**e**); the banana roots infected by *Foc*4: (**f**–**j**). The immunolabelling observed before the treatment (**a**,**f**), 6 hours after infection (**b**,**g**), 12 hours after infection (**c**,**h**), 24 hours after infection (**d**,**i**) and 48 hours after infection (**e**,**j**) are presented. Bars represent 50 µm.

JIM7 binds to methyl-esterified oligouronide epitopes in HGs whose degree of methyl esterification ranges between 35% and 81% (high methyl-esterified HGs). Except for epidermis, the distribution of JIM7 pectin covered the whole section evenly before the roots were infected with *Foc* (Fig. 5a,f). The epitope was tapering off with time after the samples were treated with *Foc*. Nonetheless, the signals were higher in roots infected with *Foc*1 than those in roots inoculated with *Foc*4 (Fig. 5a–j; see Supplementary Fig. S4). In addition, the label of roots inoculated with *Foc*4 reduced significantly in the cells enclosed by the endodermis at 48 h after infection (Fig. 5j). More roots (16.5–19.94) infected with *Foc*1 were labelled compared with roots inoculated with *Foc*4 (3.7–14.71), and the roots infected with *Foc*1 showed about twice the intension at 24 h and fourfold at 48 h after infection compared with roots inoculated with *Foc*4 (see Supplementary Fig. S4).

### JIM5

**Figure 6:**
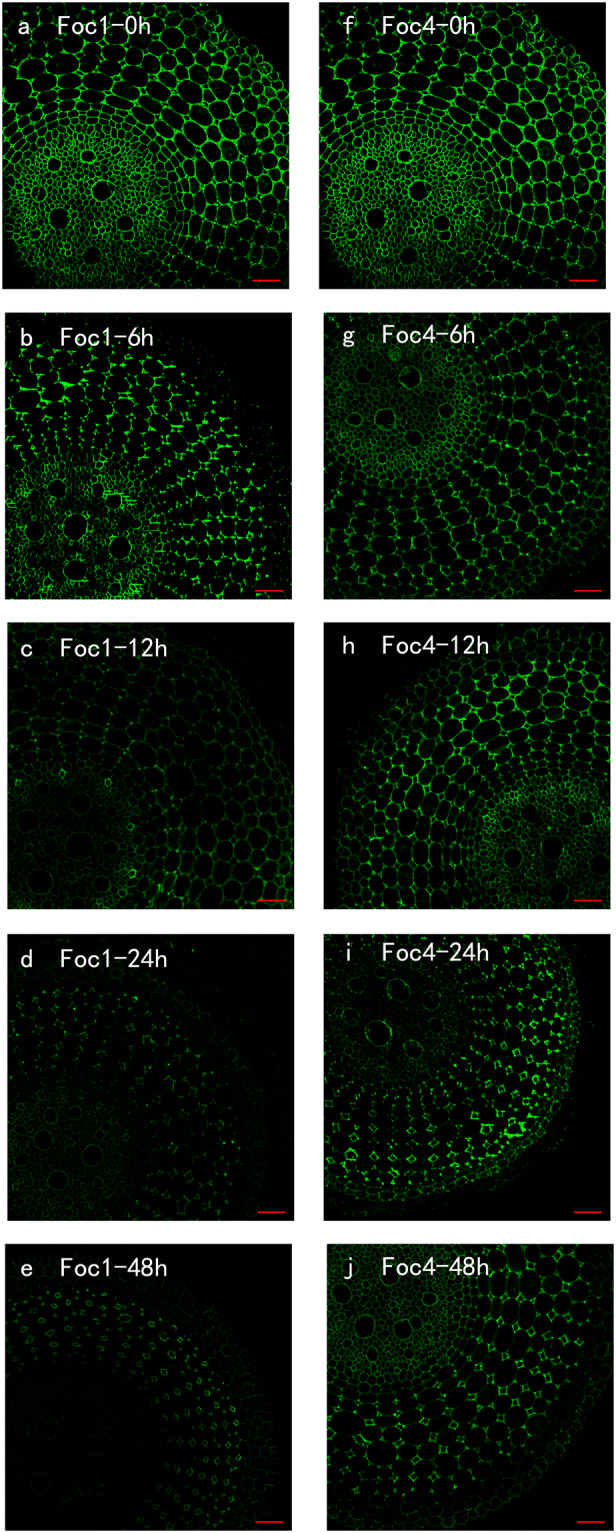
The changes in immunolocalization by JIM5 antibody in banana (*Musa* spp. AAA) roots at different hours (0, 6, 12, 24, 48 h) infection by *Fusarium oxysporum* f. sp. *cubense*. The banana roots infected by *Foc*1: (**a**–**e**); the banana roots infected by *Foc*4: (**f**–**j**). The immunolabelling observed before the treatment (**a**,**f**), 6 hours after infection (**b**,**g**), 12 hours after infection (**c**,**h**), 24 hours after infection (**d**,**i**) and 48 hours after infection (**e**,**j**) are presented. Bars represent 50 µm.

JIM5 recognises several epitopes containing 3–9 fully non-esterified oligogalacturonates^25^ within HGs with approximately 40% degree of methyl esterification^26^. The decreasing trend in roots infected with both of *Foc*1 and *Foc*4 was observed particularly in the epidermis and cells close to the epidermis; this result was similar to that observed with the monoclonal antibody JIM7. Nevertheless, the roots inoculated with *Foc*4 showed a more remarkable change compared with *Foc*1 during the entire process (Fig. 6a–j; see Supplementary Fig. S5). Prior to treatment with pathogens, the corresponding epitopes were distributed evenly all over the root section (Fig. 6a,f). At 6 h, the JIM5 pectin notably reduced in roots inoculated with *Foc*, and the change of epitope in epidermis was the most obvious (Fig. 6b,g). No differential expression was observed when the roots were infected with *Foc*4 at 12, 24 and 48 h, but significantly different results were presented by roots infected with *Foc*1, which showed continued downward trend (Fig. 6a–j). Finally, the binding of JIM5 in roots inoculated with *Foc*4 was threefold as that of roots infected with *Foc*1 at 48 h (see Supplementary Fig. S5). The roots infected with *Foc*4 exhibited a stronger signal in each time point compared with the roots infected with *Foc*1.

### 2F4

**Figure 7:**
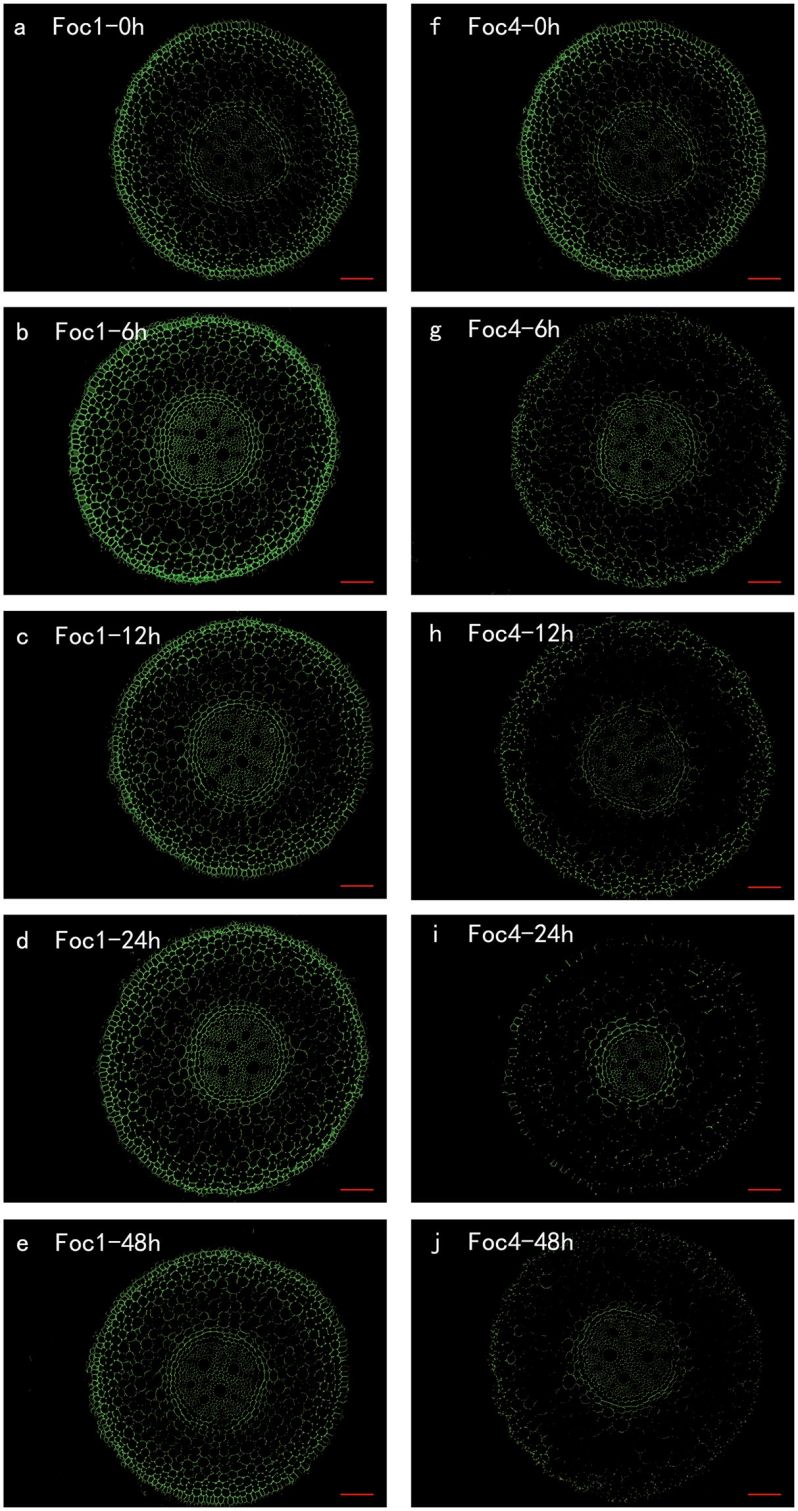
The changes in immunolocalization by 2F4 antibody in banana (*Musa* spp. AAA) roots at different hours (0, 6, 12, 24, 48 h) infection by *Fusarium oxysporum* f. sp. *cubense*. The banana roots infected by *Foc*1: (**a**–**e**); the banana roots infected by *Foc*4: (**f**–**j**). The immunolabelling observed before the treatment (**a**,**f**), 6 hours after infection (**b**,**g**), 12 hours after infection (**c**,**h**), 24 hours after infection (**d**,**i**) and 48 hours after infection (**e**,**j**) are presented. Bars represent 100 µm.

2F4 antibody recognises a conformational dimeric HGs epitope induced by a given ionic fraction between calcium and sodium. The dimer consists of two sequences of at least nine non-esterified GalA crosslinked with calcium ions, that is, the ‘egg-box’ structure^27^, which is assumed to induce gel formation and thus strengthen the cell wall^27^. The signals, recognised by 2F4, mainly concentrated on the cells of epidermis and endodermis (Fig. 7a–j). After 6 h infection with *Foc*1, the root sections contained a large amount of antigen epitopes (Fig. 7b). On the contrary, the corresponding epitopes of the roots inoculated with *Foc*4 decreased significantly at 6 h (Fig. 7g). The 2F4 labelling of the roots infected with *Foc*1 and those inoculated with *Foc*4 showed a similar tendency, where the intension decreased slowly from 12 h to 48 h (Fig. 7c–e and h–j). The main difference was that the signals of the roots infected with *Foc*1 were higher than those of the roots inoculated with *Foc*4 (Fig. 7e–j).

### Differences in *Foc* growth over time

**Figure 8:**
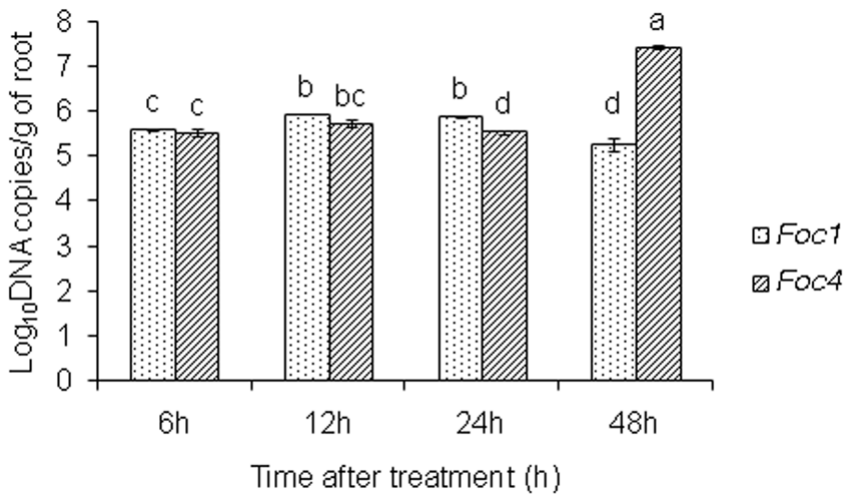
The dynamic changes of *Foc*1 and *Foc*4 of *Fusarium oxysporum* f. sp. *cubense* in banana (*Musa* spp. AAA) roots at different hours after infection. Data represent an average of three replicates ± SD. Values followed by the same letter are not significantly different using Duncan’s multiple range test at P < 0.05 after angular transformation of the data.

We analyzed different rates of fungal growth in plants inoculated with *Foc* in different time-points by qPCR with special *Foc* primer sets (Fig. 8). Our results showed that the levels of *Foc*1 was not significant different than that of *Foc4* in roots at 6 h and 12 h, but afterward *Foc*4 had greater increases in growth, while levels of *Foc*1 slowly decreased after 24 h infection.

### The induction of defence-related genes

**Figure 9:**
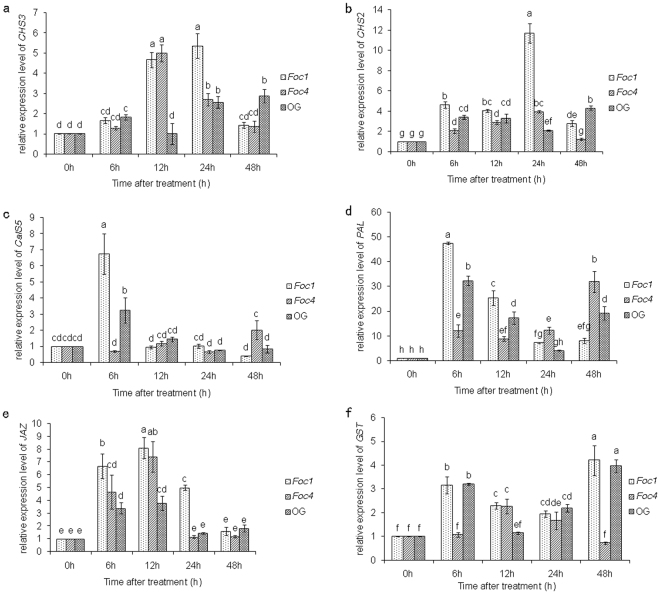
The transcriptional expression of six defence genes treated by OG, *Foc*1 and *Foc*4 of *Fusarium oxysporum* f. sp. *cubense* in banana (*Musa* spp. AAA) roots at different hours after infection. (**a**) The relative expression level of *CHS*3 treated by OG, *Foc*1 and *Foc*4. (**b**) The relative expression level of *CHS*2 treated by OG, *Foc*1 and *Foc*4. (**c**) The relative expression level of *CalS*5 treated by OG, *Foc*1 and *Foc*4. (**d**) The relative expression level of *PAL* treated by OG, *Foc*1 and *Foc*4. (**e**) The relative expression level of *JAZ* treated by OG, *Foc*1 and *Foc*4. (**f**) The relative expression level of *GST* treated by OG, *Foc*1 and *Foc*4. Data represent an average of three replicates ± SD. Values followed by the same letter are not significantly different using Duncan’s multiple range test at P < 0.05 after angular transformation of the data.

Because there were differences in the expression levels of PME, we hypothesized that mediate increase of PME may promote the production of OG, and excessive PME may result in the collapse of cell wall. To investigate this, by RT-qPCR, we measured the transcriptional levels of six defence-related genes respectively, which encode Chalcone synthase 2 and 3 (*CHS*2, *CHS*3), Callose synthase 5 (*CalS*5), phenylalanine ammonia-lyase (*PAL*), Jasmonate ZIM-domain protein (*JAZ*) and glutathione-*S*-transferase (*GST*) (Supplementary Table S1). Our results showed that all of six genes were induced significantly by OG, *Foc*1 and *Foc*4 respectively, but the pattern of induction was different (Fig. 9). Because the obvious difference of these gene expression levels in the plants treated with sterile ddH_2_O at every time point was not discovered, the samples treated with *Foc* at 0 h were utilized as the control to analyse the data. In all measured genes, the increases, induced by *Foc*1, were considerable higher than by *Foc*4 at most time points. The results suggested that OG could indeed induce the defence responses in banana, and the difference of OG accumulation might be one of influence factors on the pathogenicity difference between *Foc*1 and *Foc*4. In consideration of PME activities and PME expression levels, we concluded that appropriate increases of PME promoted the accumulation of OG, but overdoses on PME content resulted in the rapid necrocytosis.

## Discussion

A positive relationship exists between the production of enzymes by *Fusarium* spp. and its virulence, symptoms of disease or degrees of damage^28,29^. PMEs are related to the location of the pectin in the cell wall^30^, and thus they may play an important role in the development of disease.

In dicotyledonous plants and non-graminaceous monocots, pectin is a major plant cell wall polysaccharides^31^, and HGs are the main component of pectin. Pectin structure and DMs are involved in plant defence^9,13,32^. Lionetti *et al*. indicated that the pectin DMs differing between resistant and susceptible wheat genotypes possibly were related to the resistance against a fungal necrotroph^33^. Ma *et al*. reported that the spatial distributions of PMEs and different DMs of pectin were involved in the susceptibility of two different resistant banana cultivars to pathogens^24^. However, no reports addressed the different pathogenicity of *Foc*1 and *Foc*4 using histological observations. In the present study, we aimed to understand how PMEs play the role in the interaction of host and different pathogenic races of *Foc*.

In addition to roles in remodelling the plant cell wall during growth and development, PMEs participate in the pathogenic mechanism of pathogens to plant. In *Arabidopsis*, *AtPME3* is induced upon infection with *Botrytis cinerea* and *Pectobacterium carotovorum* and its expression becomes a susceptibility factor towards both microbes^13^. However, a decrease in PME expression was measured in flax (*Linum usitatissimum* L.) upon infection with *Fusarium* ^5^. We showed that two pathogenic races of *Foc* with different types of pathogenicity, induced variation in the degree of PMEs activity in the host plant. Specifically, PME activities were lower in the banana infected with *Foc*1 compared with those infected with *Foc*4. Moreover, an increase in expression of *MaPME1* was observed after infection by *Foc*. In this study, the PME monoclonal antibodies against purified total PME protein of banana were used. Due to the differences of structures and characters of PME between fungus and plant^34,35^, we speculated that the PME monoclonal antibodies are specified against plant PMEs and not those produced by *Foc*. Combining the observations of PMEs with observations of *Foc1* and *Foc4* growth in the roots at different time points (Fig. 8), we considered that fungal growth might not be responsible for alterations in PME activity in these samples. Based on these findings, we believed that the plant PME activities in roots infected by *Foc4* were higher than that of the roots infected by *Foc1*, although it was possible that *Foc*1 and *Foc*4 had different ability to produce PMEs as controlled by differences in their genetic makeup, or that expression patterns or activities of specific fungal PMEs were different between the races. Ma *et al*. also reported high increase of PME activities in the susceptible banana cultivar against *Foc*4^24^. Other pectin-degrading enzymes only degrade pectin possessing to a certain level of de-methyl esterification^10^. Thus, the increased PME activities improve the cell wall accessibility to other CWDEs, and consequently accelerate cell wall damage and indirectly promote the production of small pectic fragments, namely OGs, which can activate the defence response of host. Additionally, structural requirements for the highest elicitor activity of OGs include a low DM and a size between 10 and 15 GalA residues^36,37^. Our results showed that OGs induced the expression of defence-related genes, which were induced in plants infected with *Foc*, and the degrees of induction of the defence genes were higher in plants infected by *Foc*1 than *Foc*4 at most time-points (Fig. 9). De-methylesterified pectin is a preferred substrate of pectin degrading enzymes, and should result in increased production of OGs. Further, previous study suggested that de-methylesterified OGs were more likely to bind Ca^2+^ ^38^. However, in our results, the DM of pectin and the induced degree of these defence genes were lower in roots infected with *Foc*4 than that of *Foc*1. These results might indicate that the increase of PME activities promoted the degradation of pectin. However, it was not confirmed that pectin fragments which were excessively degraded by CWDEs were active OGs. In light of our other results, we speculated that most of these degraded smaller fragments in roots infected with *Foc*4 may have been inactive. In addition, we observed that the roots infected with *Foc4* at 72 h post-inoculation had rot and were so soft that the paraffin section could not obtained (data not shown). Therefore, we speculate that the induced mild increase of PME by pathogens promotes the production of OGs, which induce defence responses, and as a result of decreased the pathogenicity of *Foc*1. However, the excessive level of PME activities may have resulted in the rapid collapse of cell walls in banana plants infected with *Foc*4.

Marty *et al*. found an additional PME isoform in potato cultivars susceptible to *Erwinia carotovora* ^39^. Lionetti *et al*.^33^ and Zega & D’Ovidio^40^ indicated that the identification of specific PME isoforms specifically were repressed or induced in resistant or susceptible wheat cultivars to Fusarium head blight, respectively. Multiple enzyme isoforms and PMEIs could finely regulate PME activity^35^. In addition, Bethke *et al*. suggested that not total activity, but some specific effort of PMEs determined the pathogenicity of *Pseudomonas syringae* in Arabidopsis^10^. However, only one isoform *MaPME*1 had been studied in banana. Therefore, it is not clear that which isoforms involve in the process of infection. Plant PMEs catalyse the specific demethylesterification of HGs within plant cell walls, thereby releasing methanol, protons and the remaining pectin with negatively charged carboxyl groups^41^.

The factors involved in the pathogenic differences of *Fusarium* on banana are uncertain. Demethylesterification of HGs by PMEs and subsequent split by other pectinases might result in the accumulation of OG to induce the defence response, or decrease in physical strength and rigidity of the cell wall barrier, which favored pathogen invasion.

In this study, we took advantage of several cell wall antibodies to analyse the methylesterification of HGs. LM20 and JIM7 recognise highly methyl-esterified HGs. LM19 and JIM5 bind preferentially to un-esterified HGs. 2F4 are specific for a dimeric association of pectin chains, and not for isolated chains or multimeric associations^27^. However, LM19 can recognise to all pectins (pectin treated with an *Aspergillus niger* PME or with orange peel PME), showing little discrimination in relation to the extent or pattern of methyl-esterification. It was in contrast to JIM5 which bound considerably weakly to P16 (pectin treated with orange peel PME until 16% esterification remained). In contrast to JIM7, LM20 showed reduced recognition of F19 (pectin treated with an *Aspergillus niger* PME until 19% methyl-esterification remained)^42^. While there is some overlap in the functionality of these antibodies, each one has unique features. Immunofluorescence mapping indicated that pectic epitopes recognised by LM20 and LM19 antibodies presented distinct localisations at the same time points. High methyl-esterified HG domains, recognised by LM20, were detected in roots of cortical cells, whereas low methyl-esterified HG, corresponding to LM19 antibody, distributed evenly in the roots. Unlike the pectic epitopes recognised by LM20 and LM19, pectic epitopes, recognised by JIM7 presented a similar localisation with pectic epitopes recognised by JIM5. Compared with controls, no obvious differences were observed in the HGs corresponding to LM20 antibody at 48 h after infection in roots infected with *Foc*1, and considerable reductions were respectively observed in the HGs recognised by LM19, JIM5 and JIM7 antibodies. At 6 h after infection, increased PME activity in the roots inoculated with *Foc*1 was in accordance with decreased DMs. The following decrease of PME activities might result in the restoration of HGs corresponding to LM20 antibody. These findings suggested that PME activities regulate the change of pectin components, specifically those recognised by LM20 antibody, which are likely to determine the non-pathogenicity of *Foc*1 on ‘Brazilian’ banana.

Additionally, we compared the mapping difference of the roots inoculated with *Foc*1 and *Foc*4. Higher PME activities were observed in the roots infected with *Foc*4 than those infected with *Foc*1. This result was consistent with the weak signals of LM20 and JIM7, which recognised highly methyl-esterified HGs. Furthermore, a continued decrease was observed in the labelling of LM19, JIM7 and JIM5 in roots inoculated with *Foc*. The binding of LM20 and JIM7 was lower but that of LM19 and JIM5 was higher in the roots infected with *Foc*4 than in those infected with *Foc*1. Besides that, measurement of pectin DMs with colorimetry indicated that the DM of the roots infected with *Foc*4 was lower than that with *Foc*1. These results might suggest that the roots infected with *Foc*1 contained a large amount of highly methyl-esterified HGs and relatively high pectin DMs, although all treatments were carried out at the same time points. On the basis of these results, we concluded that a high abundance of highly DMs of HGs and relatively high pectin DMs might result in the low pathogenicity of *Foc*1 on banana. In addition, the high DM of pectin, which cannot be degraded by other CWDEs, possibly influenced the nutrient supply for *Foc*1.

During the interaction between host and pathogen, many reports showed that decrease of HGs recognised by JIM5 and JIM7 is involved in the response of most plants to the pathogen^15,43,44^, but the increase of JIM7 labelling is also reported^14^. Increase of HGs recognised by LM19 is a common response of many hosts to the pathogens^10^. However, an opposite result was obtained in our study, and it might be related with the content of other cell wall degrading enzymes. Unlike the decrease of the other plants^10,45^, the variation tendency of LM20 in the roots inoculated with *Foc*1 was an inverted-bell type, that is, increasing levels after an initial decrease, but the research of Ma *et al*. suggested that the binding of LM20 was increased at 6 h during the interaction between resistant banana and Foc4^24^. Although it is not clear why the binding of LM20 increased at a certain time point during the host and *Foc1*, the present results provided some information on the dynamic changes of PMEs, pectin DMs and individual HG levels, thereby suggesting that the difference of sampling timing, plant species and pathogens might result in the differences of results.

The activities of PMEs on pectin can result in two major mechanical outcomes: low DM pectin is a target for polygalacturonases, which cleave it further and likely intensify gel softness; low DM pectin is also ready to cross-like with Ca^2+^ to increase gel rigidity^44^. HGs with sufficiently low DMs (<40%) and at least nine consecutive un-esterified GalA residues can interact with Ca^2+^, which result in calcium bridges also known as ‘egg-box’ structure^27^.

The antibody 2F4 is specific to calcium crosslinked HGs, and these bridged HGs might be more resistant to fungal invasion than unbridged epitopes^46^. These 2F4 antibodies are specific for a dimeric association of pectin chains such as the one described as the ‘egg-box’ model, and not for isolated chains or multimeric associations^27^. However, we did not clear the ratio of a dimeric association of pectin chains. Therefore, we indicated that there may be no direct or inevitable correlation between the 2F4 binding and the binding of LM19 and JIM5.

The labelling of 2F4 mainly concentrated on the cells of epidermis and endodermis. In the banana-*Foc*4 interaction, the occurrence of the 2F4-epitope was gradually lower. Ma *et al*. also reported that the attack of *Foc*4 results in the decrease of 2F4 labelling^24^. Nevertheless, the labelling of 2F4 remains stable during the interaction between banana and *Foc*1, except for the increase at 6 h post infection. These results indicated that the increase of pectin recognised by 2F4 might be responsible for the non-pathogenicity of *Foc*1 on ‘Brazilian’ banana, and its decrease might be an element of the strong pathogenicity of *Foc*4.

In summary, we observed that the infection of *Foc* induced the varying degrees of PME activities, and the activity was lower level in banana inoculated with *Foc*1 than that with *Foc*4. The increase of enzyme activity might trigger the immune response induced by OG, and the excessive enzyme activity probably would result in the direct damage of cell wall. In addition, the banana infected with *Foc*1 showed higher abundance of highly DMs of HGs and relatively higher pectin DMs than that with *Foc*4. This result suggested that high abundance of highly DMs of HGs and relatively high pectin DMs might decrease the pathogenicity of *Foc*1. Therefore, the degrees of PME activities induced by pathogen and the level of highly DMs of pectin may contribute the observed difference in pathogenicity between *Foc*1 and *Foc*4. Thus, our observations provide new insights regarding how different pathogenic races in banana may differ in ability to cause disease.

## Materials and Methods

### Plant materials and pathogen inoculation

*Musa* spp. AAA cv. ‘Brazilian’ was used in our study. Banana plantlets were propagated under a sterile tissue culture condition. We previously performed inoculation experiments with *Foc* and confirmed that ‘Brazilian’ cultivar is susceptible to *Foc*4 and resistant to *Foc*1^47,48^. Tissue-cultured plants, incubated at 28 ± 2 °C under cool-white light (20 μmol m^−2^ s^−1^) on a shaker at 90 rpm, were cultured in liquid rooting medium^24^. First, the roots were induced for two weeks. Second, one root of each seedling was cut off for pathogen penetration. Third, these treated plants were transferred to a new medium, which contained *Foc*1 or *Foc*4 at a final concentration of 5 × 10^2^ ml^−1^ or contained OGs (elicityl-oligothch, France, DP: 10–15) at a final concentration of 20 μg ml^−1^. Seedlings immersed in the culture medium without the pathogen (mock inoculation) were used as a control. Afterwards, the infected roots were harvested at 6, 12, 24 and 48 h after the inoculation. All tissue samples were collected at the same time points to minimise differences. The control samples were also collected at the same time points after mock inoculation.

### Enzyme activity assays

Samples (0.2 g), washed in distilled water, were homogenised in 1.5 ml of extraction solution (0.25 M NaCl, 0.1 M acetic acid–sodium acetate buffer, pH 5.0) at low temperature. The suspensions were centrifuged at 10000 rpm for 20 min at 4 °C. Titration of generated carboxyl groups, which is a modification of the method developed by Marcus and Schejter^49^, was used to determine the PME activity in protein extracts. Aliquots of the supernatant (0.3 ml) were added to 3 ml of PME substrate (1% citrus pectin). PME activity was measured at pH 6.5 via continuous automatic titration with 0.01 M NaOH of the carboxyl groups released during the enzyme reaction. The titration volume was used to determine generated carboxyl groups analytically. A PME activity unit was defined as the number of microequivalents of carboxyl groups cleaved by 1 mg of enzyme min^−1^ at 30 °C and pH 6.5.

### Protein determination

Protein content was determined by the method of Bradford using the protein dye reagent (Sigma Chemical Co.). A calibration curve was made with bovine serum albumin (BSA)^50^.

### Alcohol insoluble residue (AIR) preparation

Plant cell wall material was prepared according to the method described by Louvet *et al*.^51^ with some modification. Briefly, AIR preparation was carried out as follows: the roots were gently cut and washed thrice with 70% ethanol (v/v) at 70 °C for 30 min. After centrifugation, the supernatant was removed. The pellet was crushed in liquid nitrogen and freeze dried.

### Measurement of pectin DMs with colorimetry

The DMs were determined via saponification of AIR and enzymatic oxidation of methanol released by alcohol oxidase. The DMs were calculated by the proportion of methanol content to galacturonic acid (GalA) molar content. The methanol assay was adopted from the methods described by Louvet *et al*.^52^ and Klavons and Bennett^53^. GalA content was determined by the metahydroxydiphenyl assay (MHDP) adapted from Blumenkrantz and Asboe-Hansen^54^. Three replicates were prepared for each treatment, and each experiment was repeated thrice. GalA was calculated as micrograms of GalA per gram of AIR.

### Quantitative real-time PCR (RT-qPCR) assay for defence-related genes

Total RNA was extracted using the QIAGEN RNeasy plant mini kit (QIAGEN, CA) and treated with RNasefree DNAse I (Promega, USA). RNA was reverse transcribed in 20 μl of reaction system using the PrimeScript^TM^ RT Master Mix Kit (TaKaRa). The sequence of *RPS2* gene was used as the reference gene^51^. Gene-specific primers designed based on the gene sequences using Primer 5.0 software. The primer sequences are listed in Table S1. The RT-qPCR was performed using the SYBR Premix Ex Taq Kit (TaKaRa) according to the manufacturer’s protocol. All RT-qPCR reactions were carried out using TP800 Thermal Cycler Dice (Takara, Japan). Standard curves based on threshold cycles for 10-fold dilution series of cDNA were obtained. A total of five 10-fold dilution steps of cDNA were run in triplicate on each well plate. PCR efficiency and correlation coefficient (R^2^) were calculated from the threshold cycles of these standard dilution steps. The reactions were run with primer pairs at annealing temperature of 57 °C. The conditions were as follows: initial holding at 95 °C for 30 s, followed by a two-step program of 95 °C for 15 s and annealing temperature for 30 s for 40 cycles. Each sample was analysed in three technical replicates. The relative changes in gene expression levels were calculated using the formula 2^−ΔΔCT^ ^55^.

### Immunofluorescence labeling

For immunolocalisation of PMEs and HGs with different DMs, the roots were treated with *Foc*1 and *Foc*4 for 6, 12, 24 and 48 h and harvested at the same time points to minimise the effect of other factors. The protocol for fixation, embedding of samples and immunolabelling was carried out as described by Xu *et al*.^56^. We labelled the sections with primary monoclonal antibodies PME, 2F4, LM19, LM20, JIM5 and JIM7. The anti-mouse lgG-FITC (F9006, Sigma) was used as secondary antibody for PME and 2F4 antibodies, and the anti-rat lgG-FITC (F6258, Sigma) was used for the other antibodies. Sections marked only with secondary antibodies were used as controls. At least three replicates were prepared for each antibody. Images were collected using a LSM 7 DUO laser scanning confocal microscope (ZEISS, Germany) and Olympus BH-2-FRCA microscope, and fluorescence signals were examined with LSM 7 DUO laser scanning confocal microscope (ZEISS, Germany) by the function “Histo” to determine an average fluorescence signal of a whole root section.

### Real-time PCR amplification and quantification for abundance of *Foc*

Fungal mycelium and *Foc*-infected banana roots were immediately frozen in liquid nitrogen and ground to fine powder. Genomic DNA (gDNA) was extracted according to Lin *et al*.^57^, and the DNA samples were dissolved in 0.1× TE buffer (1 mM Tris-HCl, pH 8; 0.01 mM EDTA). In order to detect *Foc*1 and *Foc*4 isolates, a specific primer set Foc-1/Foc-2 (5′-CAGGGGATGTATGAGGAGGCT/5′-GTGACAGCGTCGTCTAGTTCC) was used for Foc4^57^ and another specific primer set W1805F/W1805R (5′ GTTGAGTCTCGATAAACAGCAAT/5′GACGAGGGGAGATATGGTC) were used for *Foc*1^58^. A 242-bp size fragment was produced by PCR from *Foc*4 gDNA and a 354-bp from *Foc*1 gDNA. The *Foc* fragments were gel-purified, cloned into pMDTM18-T vector (TAKARA), and sequenced. After plasmid purification, the plasmid DNA concentration was quantified using spectrophotometer (NANODROP ONE, USA). The copy number of standard plasmids was calculated according to plasmid (2692 bp) plus insert lengths (242 bp/354 bp) and assuming a molecular mass of 660 Da for a base pair. Standard DNA stock solutions of 10^10^ copies of plasmid µL^− 1^ were prepared. Ten-fold serial dilutions of the target DNA, ranging from 10^8^ to 10^3^ copies per reaction, were performed for standard curve plotting and melting-curve analysis of real-time PCR amplification and yielded linear and reliable results (correlation coefficient, R2 > 0.99).

Next, the real-time PCR assay was used to detect *Foc* in *Foc*-infected banana. The *Foc*-infected banana samples were surface-sterilized with 0.1% Clorox bleach for 1 min, rinsed with sterile water three times, and airdried. The surface-sterilized dried samples were picked for PCR amplification. The tested DNA samples were extracted from roots for RT-qPCR assays. When testing DNA samples, we used *RPS2* gene as the reference gene to make samples to be normalized.

### Statistical analysis

Statistical analyses were carried out using ANOVA by the statistical program SPSS 13.0 for Windows (SPSS Inc., Chicago, IL, USA). At least three replicates were used in the experiments. Data are expressed as the mean ± SD. Multiple differences among means were evaluated using Duncan’s multiple range tests at a 5% probability level.

## Electronic supplementary material

 Supplementary Information
