# Supplementary material for: Pectin methylesterases contribute the pathogenic differences between races 1 and 4 of Fusarium oxysporum f. sp. cubense
Source: Sci Rep. 2017 Oct 13;7:13140. doi: 10.1038/s41598-017-13625-4 (PMC5640671; doi:10.1038/s41598-017-13625-4)
Supplement: Supplementary file 1 — Supplementary Information [file 41598_2017_13625_MOESM1_ESM.pdf]

Pectin methylesterases contribute the pathogenic differences between races 1 and 4 of *Fusarium oxysporum* f. sp.*cubense*

Huiyun Fan[1], Honghong Dong[1], Chunxiang Xu[2], Jing Liu[2], Bei Hu[2], Jingwen Ye[1], Guiwan Mai[1], Huaping Li[1]*

[1]State Key Laboratory of Conservation and Utilization of SubtropicalAgro-bioresources, Guangdong Province Key Laboratory of Microbial Signals and Disease Control, College of Agriculture, South China Agricultural University, Guangzhou 510642, China.

[2] College of Horticulture, South China Agricultural University, Guangzhou 510642, China;

*Correspondence to huaping@scau.edu.cn

Table S1 Primers for quantitative real-time PCR

Gene	GeneBank number	Primer sequences (Forward/ Reverse)	Product size (bp)
MaPME1	FJ264505.1	GCTACCAAGACACCCTCTAC<br>GTACAAGTTGCAGTTCTGGAGC	84
RPS2	HQ853246.1	TAGGGATTCCGACGATTTGTTT<br>TAGCGTCATCATTGGCTGGGA	122
CHS2	XM_009405523.2	GCATCACCAGGAGCAACCAC<br>CATGTACGCGCCGATGTTAG	137
CHS3	XM_009406457.2	CGGCAGGTGCTGAGCGAGTA<br>GTGGTTGCCTTTCCGTCCTC	101
CalS5	XM_018828485.1	GACCTTATAAGTGACAGGAAAGAGATGG<br>CACAGATGCGTTTCCACAGATCAGAATC	175
PAL	XM_009419417.2	ACATATCCTAAGAAGCCCCACCACC<br>TCGTCAAAGTGGGCACAAGAGAG	206
JAZ	XM_009411294.2	TGCAAGAGGTTCATCATAGGACAGT<br>TCTTCAGACATGGAAGTGGCCTC	198
GST	XM_009409198.2	CCTTCAGATGTTCTTCCAGTGCATG<br>TGTTATCACGGCGATCATTGAGGAG	185

Table S2 Antibodies recognizing different HGs and their antigens

Antibody	Antigen	Secondary antibody
JIM5[1]	Partially methyl-esterified HG epitope: unesterified and partially esterified residues (up to 40%)	Anti-rat-IgG-FITC
JIM7[1]	Partially methyl-esterified HG epitope: methyl-esterified residues (up to 80%)	Anti-rat-IgG-FITC
LM19[2]	Low methyl-esterified HG	Anti-rat-IgG-FITC
LM20[2]	Highly methyl-esterified HG	Anti-rat-IgG-FITC
2F4[3]	Un-esterified/Calcium ion cross-linked HG	Anti-mouse-IgG-FITC

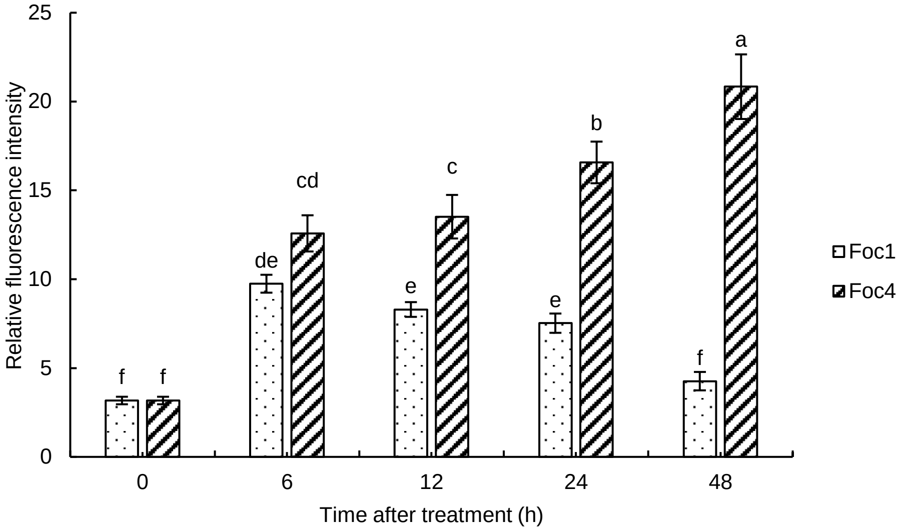

Figure S1 The relative fluorescence intensity of PME in banana (*Musa* spp. AAA) roots after infection with *Fusarium oxysporum* f. sp.*cubense*. *Foc*1: *Fusarium oxysporum* f. sp.*cubense* race 1; *Foc*4: *Fusarium oxysporum* f. sp.*cubense* race 4. For quantification of fluorescence signal, the mean relative fluorescence intensity (n =3 sections of roots) was measured with ZEN 2012 software.

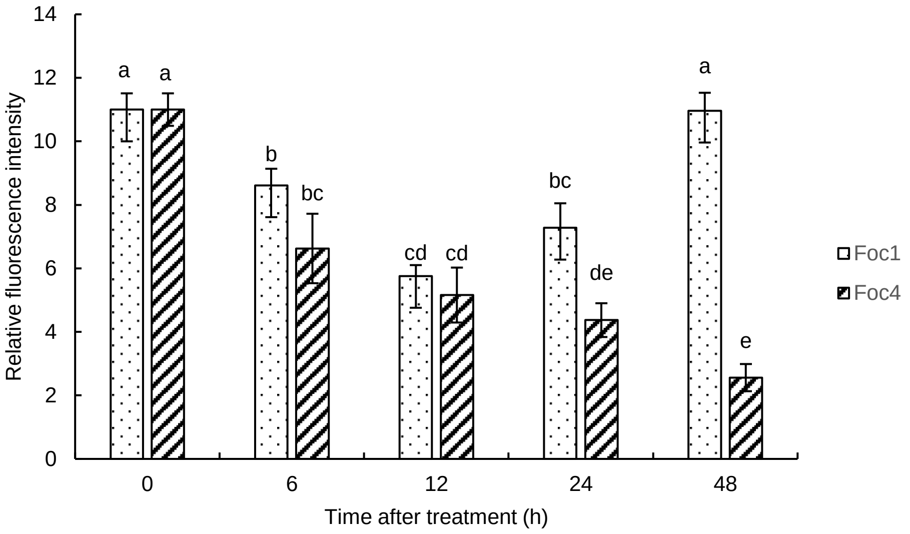

Figure S2 The relative fluorescence intensity of pectins recognized by LM20 in banana (*Musa* spp. AAA) roots after infection with *Fusarium oxysporum* f. sp.*cubense*.

*Foc*1: *Fusarium oxysporum* f. sp.*cubense* race 1; *Foc*4: *Fusarium oxysporum* f. sp.*cubense* race 4. For quantification of fluorescence signal, the mean relative fluorescence intensity (n =3 sections of roots) was measured with ZEN 2012 software.

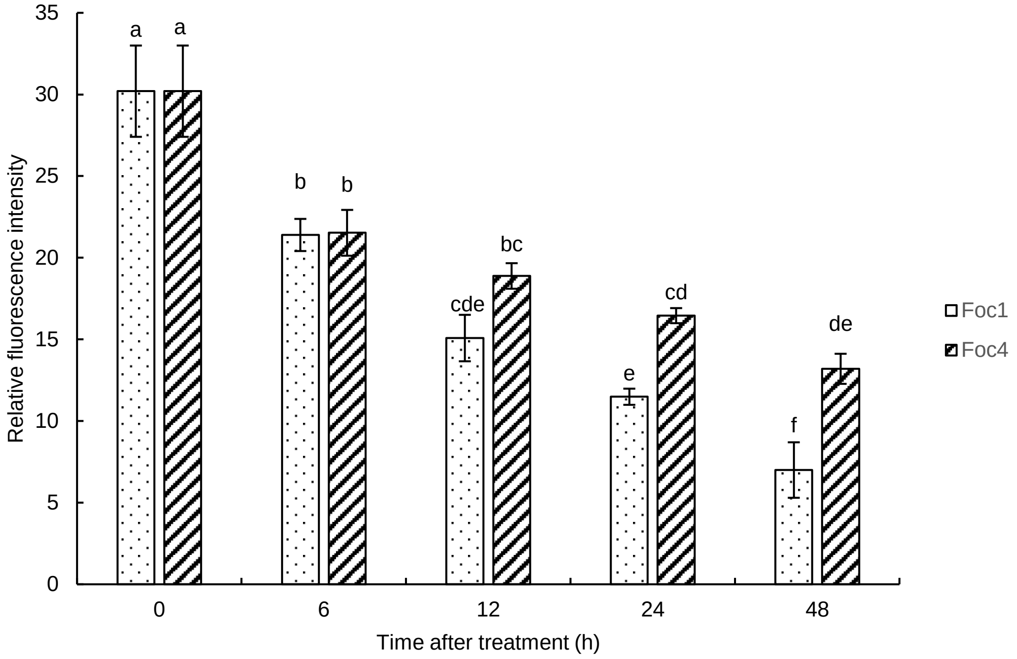

Figure S3 The relative fluorescence intensity of pectins recognized by LM19 in banana (*Musa* spp. AAA) roots after infection with *Fusarium oxysporum* f. sp.*cubense*.

*Foc*1: *Fusarium oxysporum* f. sp.*cubense* race 1; *Foc*4: *Fusarium oxysporum* f. sp.*cubense* race 4. For quantification of fluorescence signal, the mean relative fluorescence intensity (n =3 sections of roots) was measured with ZEN 2012 software.

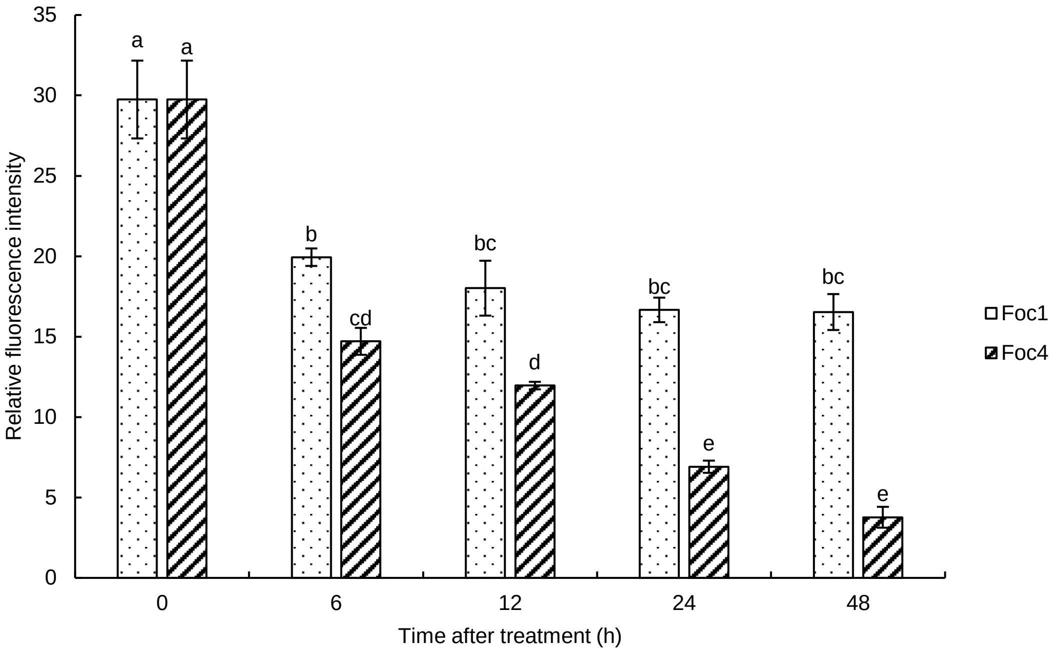

Figure S4 The relative fluorescence intensity of pectins recognized by JIM7 in banana (*Musa* spp. AAA) roots after infection with *Fusarium oxysporum* f. sp.*cubense*.

*Foc*1: *Fusarium oxysporum* f. sp.*cubense* race 1; *Foc*4: *Fusarium oxysporum* f. sp.*cubense* race 4. For quantification of fluorescence signal, the mean relative fluorescence intensity (n =3 sections of roots) was measured with ZEN 2012 software.

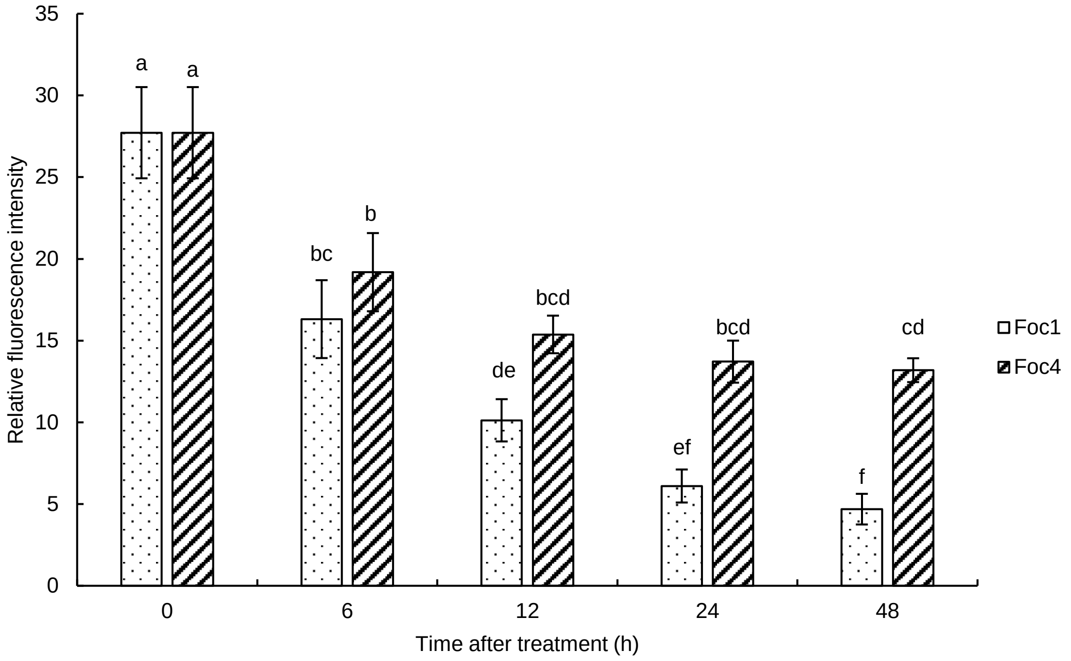

Figure S5 The relative fluorescence intensity of pectins recognized by JIM5 in banana (*Musa* spp. AAA) roots after infection with *Fusarium oxysporum* f. sp.*cubense*.

*Foc*1: *Fusarium oxysporum* f. sp.*cubense* race 1; *Foc*4: *Fusarium oxysporum* f. sp.*cubense* race 4. For quantification of fluorescence signal, the mean relative fluorescence intensity (n =3 sections of roots) was measured with ZEN 2012 software.

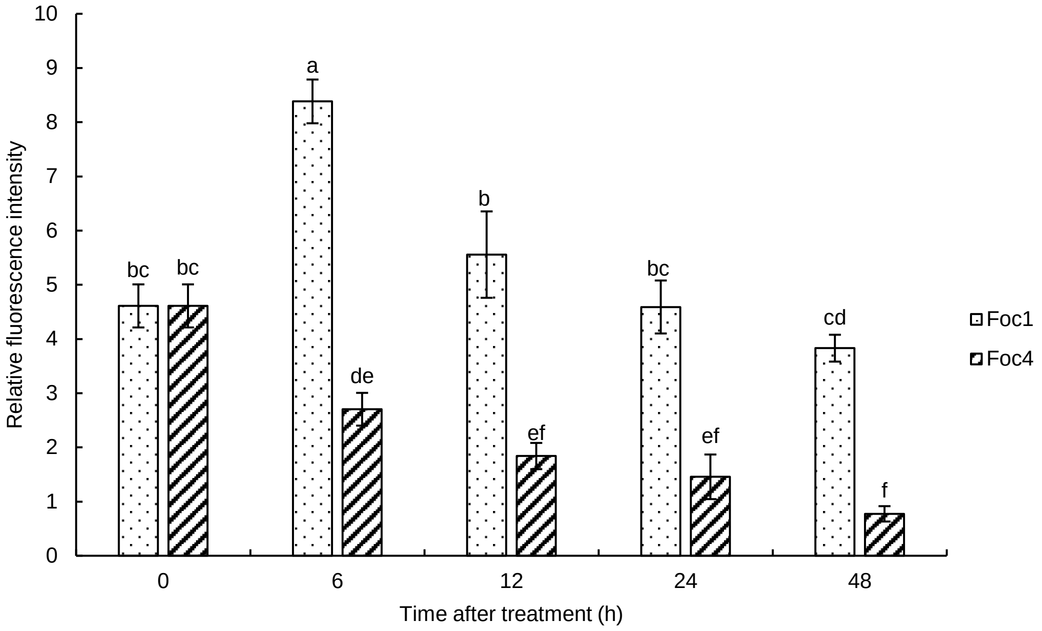

Figure S6 The relative fluorescence intensity of pectins recognized by 2F4 in banana (*Musa* spp. AAA) roots after infection with *Fusarium oxysporum* f. sp.*cubense*.

*Foc*1: *Fusarium oxysporum* f. sp.*cubense* race 1; *Foc*4: *Fusarium oxysporum* f. sp.*cubense* race 4. For quantification of fluorescence signal, the mean relative fluorescence intensity (n =3 sections of roots) was measured with ZEN 2012 software.

References:

1. Knox, J.P., Linstead, P.J., King, J., Cooper, C. & Roberts, K. Pectin esterification is spatially regulated both within cell walls and between developing tissues of root apices. *Planta* (1990).

2. Verhertbruggen, Y., Marcus, S.E., Haeger, A., Ordaz-Ortiz, J.J. & Knox, J.P. An extended set of monoclonal antibodies to pectic homogalacturonan. *Carbohydrate Research* **344**, 1858-1862 (2009).

3. Liners, F.O., Letesson, J.J., Didembourg, C. & Cutsem, P.V. Monoclonal Antibodies against Pectin. *Plant Physiology* **91**, 1419-24 (1989).
